# Prevalence, Risk Factors, and Health Sequelae of Domestic Violence for Females During Reproductive Age: A Community-Based Cross-Sectional Study

**DOI:** 10.3390/nursrep16020060

**Published:** 2026-02-11

**Authors:** Randa Mohamed Abobaker, Fares Hameed D. Alshammari, Nabila Salem Mohamed, Rania Ahmed Elbasiony, Naima Mohammed Elsayed, Amna Nagaty Aboelmagd, Faisal Khalaf Alanazi, Hammad Ali Fadlalmola, Amal Hashem Mohamed

**Affiliations:** 1 College of Nursing, University of Hafr Al-Batin, Hafr Al-Batin 39524, Saudi Arabia; fhalshammari@uhb.edu.sa; 2 Faculty of Nursing, Zagazig University, Zagazig 44519, Egypt; nabilahussien1012@gmail.com (N.S.M.); naimamohammed@nec.edu.sa (N.M.E.); 3 Nursing Department, North Private College of Nursing, Arar 73222, Saudi Arabia; amalhasem@nec.edu.sa; 4 Nursing Department, Mohammad Al-Mana College for Medical Sciences, Dammam 34222, Saudi Arabia; r.elbasiony@machs.edu.sa; 5 Faculty of Nursing, Minia University, Minia 61519, Egypt; amnanagati@mu.edu.eg; 6 Nursing Department, Al-Ghad College for Applied Medical Sciences, Madinah 42351, Saudi Arabia; 7 College of Nursing, Northern Border University, Arar 73222, Saudi Arabia; faisal.alanazi@nbu.edu.sa; 8 College of Nursing, Taibah University, Madinah 42353, Saudi Arabia; hazzminno345@gmail.com

**Keywords:** domestic violence, reproductive age, women, prevalence, risk factors, health consequences, Egypt, cross-sectional study, nursing

## Abstract

**Background/Objectives**: Domestic violence against women is a widespread global health issue profoundly affecting victims, their families, and society. This study aimed to assess the prevalence, patterns, risk factors, and health sequelae of domestic violence among females during reproductive age in Sharkia governorate, Egypt. **Methods**: A cross-sectional descriptive study was conducted from April to December 2024. A total of 379 females of reproductive age (15–49 years) were recruited using simple random sampling from secondary schools and Maternal and Child Health centers affiliated with the Ministry of Health. Data were collected using a structured interview questionnaire covering sociodemographic characteristics, violence exposure (physical, psychological, economic, and sexual), risk factors, causes, severity, perpetrators, and consequences. **Results**: The overall prevalence of domestic violence was 88%. Psychological violence was the most common form (78%), followed by physical violence (63%), and economic violence (43%). Insults were the predominant form of verbal abuse, while slapping and beating were the most common manifestations of physical violence. Husbands were identified as the primary perpetrators across all violence types. Major risk factors included cigarette use by the abuser (47%), alcohol and drug use (14%), and psychological problems (11%). The most frequently reported consequences were anxiety, fear, and depression (82%), followed by insomnia (55%) and seeking separation (49%). **Conclusions**: Domestic violence against women of reproductive age is highly prevalent in the study setting, with significant physical and psychological consequences. Comprehensive interventions, including awareness campaigns, legal enforcement, women empowerment programs, and healthcare provider training, are urgently needed to address this critical public health issue.

## 1. Introduction

Violence against women is a major problem globally, particularly in developing countries, representing one of the most systematic and widespread human rights violations. It is rooted in gendered social structures rather than individual and random acts, cutting across age, socioeconomic, educational, and geographic boundaries while affecting all societies. It remains a major obstacle to achieving gender equality and eliminating discrimination globally [1,2].

The World Health Organization (WHO) has reported that more than one quarter of women of reproductive age (15–49 years) worldwide experience physical violence by their intimate partners at least once in their lifetime [3]. Globally, over 1.3 million people die each year from all forms of violence, accounting for 2.5% of global mortality, making it the fourth leading cause of death worldwide [4]. In Egypt, the 2021 Demographic and Health Survey reported that 43.8% of women experienced violence. Psychological violence was the most prevalent type (96.0%), followed by sexual violence, which was the least common (13.5%) [5,6].

Violence can take various forms, including physical, sexual, emotional, and economic abuse, and occurs in different settings involving family members, friends, or other familiar individuals [7]. Recent estimates suggest that between 10% and 53% of ever-partnered women have experienced physical violence by an intimate partner [8]. Risk factors for violence include young age, poor mental health, low self-esteem, a history of physical discipline during childhood, marital instability, poverty-related issues, and low levels of community intervention against violence [9].

In 2017, the Committee on the Elimination of Discrimination Against Women (CEDAW) asserted that violence against women is a form of discrimination and elaborated international standards on gender-based violence in General Recommendation No. 35. The CEDAW Committee recognized that the prohibition of gender-based violence against women has evolved into a principle of customary international law, binding all states [10].

Today, violence against women affects one in every three women (30%) globally in their lifetime. It is present in every country without exception and crosses boundaries of culture, class, education, income, and race [11,12]. Despite the magnitude of literature on this problem, the field lacks a comprehensive and homogeneous way to measure and compare the extent of violence against women across countries. Proper quantification is essential for developing preventive policies and strategies to address this pervasive issue [13].

In the context of Middle Eastern countries, and particularly in Egypt, cultural norms and traditional gender roles may contribute to underreporting and the normalization of violence against women. Understanding the local patterns, risk factors, and consequences of domestic violence is crucial for developing culturally appropriate interventions and policies.

Today, there is a great importance for adequately training maternity nurses to deal with violence against women and improve the quality of care. Additionally, strategies must be implemented to ensure that all human rights and fundamental freedoms of women and girls are protected and that all forms of discrimination are eliminated. Every form of violence threatens all women and girls and limits their ability to make choices about their lives. It is a serious human rights violation and a public health problem of global scope. It contains all the ways our society realizes and oppresses women. So that helps the woman formulate a plan of action for either leaving or remaining safely in the relationship. Offer information about accessible resources, such as hotline and shelter numbers. Suggest she pack a quick getaway bag with personal matters to be unseen or left with a trusted neighbor or friend. Recommend she have an extra set of car keys, house keys, money, and any legal documents needed for identification.

This study aimed to assess the prevalence, risk factors, and health sequelae of domestic violence among females during reproductive age. The specific objectives of this study were: (1) to determine the prevalence and patterns of domestic violence (physical, psychological, economic, and sexual) among females of reproductive age; (2) to identify the sociodemographic and clinical factors associated with different forms of violence; (3) to explore the risk factors and causes of domestic violence from the perspectives of the affected women; and (4) to assess the health consequences and sequelae of domestic violence on the study participants.

The research question was: What is the prevalence, risk factors, and health sequelae of domestic violence for females during reproductive age at general and private secondary schools and Maternal and Child Health centers in Sharkia governorate, Egypt?

## 2. Materials and Methods

### 2.1. Study Design and Setting

This was a cross-sectional descriptive study conducted at general and private secondary schools and Maternal and Child Health centers affiliated with the Ministry of Health in Sharkia governorate, Egypt. The study was conducted from April 2024 to January 2025. These settings were selected because they represent almost all districts in the Sharkia governorate and serve clients from various socioeconomic and cultural groups. A cross-sectional descriptive design was selected because it is well-suited for determining the prevalence and patterns of domestic violence at a specific point in time, allowing for efficient data collection across a large sample while examining multiple variables simultaneously. This design is appropriate for exploratory studies that aim to identify potential risk factors and associations, particularly in sensitive research areas where longitudinal follow-up may be challenging due to participant safety concerns and ethical considerations.

### 2.2. Participants and Sampling

The study participants were females of reproductive age (15–49 years) recruited through simple random sampling from Zagazig Secondary School, Gamal Abdel Nasser Secondary School for Girls in El Zagazig city, and Maternal and Child Health centers affiliated with the Ministry of Health. Written approval was obtained from school directors, and confidentiality was maintained. Data were collected from mothers and daughters separately to ensure privacy, after explaining the study objectives and obtaining informed consent.

The sample size was calculated using the formula for finite populations with a 95% confidence level, margin of error (α) of 0.05, effect size (d) of 0.5, and statistical power of 0.80 using G*Power analysis (Version 3.1.9.7). The target population comprised 4100 females of reproductive age. The sample correction for community size followed the equation: n* = n/(n/N) + 1, resulting in a sample size of 379 participants. The sample was distributed randomly, considering the relative distribution to community groups. The 379 participants included 55 female students (14.5%) aged 17–19 years, recruited from secondary schools, and 324 women (85.5%) aged 20–49 years, recruited from Maternal and Child Health centers. Among the adult participants, 168 (44.3%) were married, 91 (24.0%) were divorced, and 27 (7.1%) were widowed. For participants under 18 years of age (n = 20), parental consent was obtained prior to their participation in the study, in accordance with national research ethics guidelines.

A pilot study was conducted on 35 females of reproductive age to test the feasibility, clarity, and objectivity of the data collection tool. Necessary modifications, corrections, and additions were made based on the pilot study results. The participants in the pilot study were excluded from the final sample.

### 2.3. Data Collection Tool

Data were collected using a structured interview questionnaire developed by the researchers A structured interview questionnaire was chosen as the data collection method for several reasons: (1) it allows for standardized data collection ensuring consistency across all participants; (2) face-to-face interviews enable clarification of questions and provide a supportive environment for discussing sensitive topics such as domestic violence; (3) this method accommodates participants with varying literacy levels; and (4) structured interviews have demonstrated high reliability in previous violence research in similar cultural contexts. It was prepared by the researcher based on the recent related literature review and experts’ opinions [14], which included the following components:

#### 2.3.1. Part 1: General and Clinical Characteristics (14 Items)

This section consisted of two subsections:

- Sociodemographic characteristics (10 items): age, marital status, marital age, education level, place of residence, occupation, husband’s age, husband’s occupation, husband’s education, and monthly income.
- Clinical characteristics (4 items): number of pregnancies, births, abortions, and living children.

#### 2.3.2. Part 2: Women’s Exposure to Different Forms of Violence

This section assessed female exposure to physical, psychological, economic, and sexual violence through specific questions about the prevalence and manifestations of each type.

#### 2.3.3. Part 3: Severity, Causes, and Perpetrators of Violence (15 Items)

This section included:

- Causes of violence (9 items): lower income, habits and traditions, embedded violence, illiteracy, unemployment, drug use, variety of social education status, tolerance of violence, female neglect, and family involvement.
- Severity indicators (6 items): destruction of property, control of money, burning, attack with a weapon or object, pressure to have sex, and increased frequency of violence.
- Perpetrators (6 categories): husband, husband’s family, husband, and family together, father, mother, father, and mother together.

#### 2.3.4. Part 4: Risk Factors of Abuse (4 Items)

This section explored risk factors from the woman’s perspective, including cigarette use, drug and/or alcohol use, unknown factors, and psychological problems of the abuser.

#### 2.3.5. Part 5: Violence Consequences (11 Items)

This section assessed the impact of violence, including physical pain, anxiety, fear, depression, physical impairment, suicidal thoughts, hospitalization, work absence, school absence, stopping eating, obesity, insomnia, and seeking separation.

### 2.4. Data Collection Procedure

Formal consent was obtained from all participants after they were informed of the study’s nature and aims. The researchers introduced themselves to potential participants and provided a full explanation of the study objectives to obtain their acceptance and cooperation. Participants were informed of the voluntary nature of the study and their right to withdraw at any time.

After providing informed consent, each female participant underwent a face-to-face structured interview lasting 30–40 min at a health center or another location preferred by the participant. Each female was interviewed separately to provide her with the opportunity to discuss her experience of violence freely and confidentially.

### 2.5. Validity and Reliability

The data collection tools were reviewed by three experts in maternity nursing and community health nursing to test content validity. The tools were modified according to experts’ suggestions to ensure clarity, relevance, and comprehensiveness. The researchers used reliability to verify the instruments’ internal coherence. The experts evaluated the tool for clarity, relevance, inclusiveness, applicability, and ease of understanding. Cronbach’s alpha coefficients evaluated the reliability of the tool items. The results demonstrated high reliability: 0.842 for the women’s knowledge assessment questionnaire about Prevalence, risk factors, and 0.866 for causes, severity, and health sequelae.

### 2.6. Data Analysis

After data collection, all data were coded, organized, categorized, and transferred into specially designed formats. Statistical analysis was performed using the Statistical Package for the Social Sciences (SPSS) version 28. Descriptive statistics, including frequencies, percentages, means, and standard deviations, were used to describe the study variables and sample characteristics. The Chi-square test was used to test for significant differences between actual and expected distributions. Pearson’s correlation coefficient was used to measure the range and direction of relationships between variables. A *p*-value of less than 0.01 was considered statistically significant.

### 2.7. Ethical Considerations

Ethical approval was obtained from the College of Nursing at Zagazig University in January 2024 (ID/Zu. Nur. REC: 306). Informed consent was obtained from each participant before enrollment in the study after clarification of the nature and purpose of the study. For participants under 20 years of age, parental consent was obtained. Participants were reassured about the confidentiality of the obtained information and informed about their right to withdraw from the study at any time without consequences.

## 3. Results

### 3.1. Sociodemographic and Clinical Characteristics

Table 1 presents the sociodemographic data and clinical history of the study sample and their correlation with different forms of violence. More than one-third (34.8%) of the females were between 29 and 39 years of age. The majority (51.2%) were housewives, and nearly one-third (35.3%) had secondary education. Regarding husbands, 75.7% were employed, and 42% had a monthly income of 4000 pounds or more.

A highly statistically significant correlation was found between sociodemographic variables (female age, marriage age, women’s education, occupation, residence, marital status, husband’s age, education, occupation, and income) and different forms of violence (*p* = 0.0001).

Regarding clinical history, more than half (56.7%) of the study sample had fewer than five pregnancies, 79.4% had fewer than five births, 79.4% had experienced abortion, and more than two-thirds (65.4%) had fewer than five living children.

### 3.2. Prevalence and Forms of Violence

Table 2 reveals that the overall prevalence of domestic violence was 92.6% of participants exposed to one or more forms of violence. More than three-quarters (76.6%) of the study sample were exposed to psychological violence, with yelling being the most common form (52.4%), followed by belittling or humiliation (42.5%).

Nearly two-thirds (66.1%) of the sample experienced physical violence. The most common forms were slapping, throwing, and beating (52.4%), followed by kicking, dragging, or throwing things (34.8%).

Economic violence was experienced by 63.5% of participants. The most common forms were being prevented from owning anything (39%) and being prevented from working or having their salary taken (35.3%).

Sexual violence was reported by 60.1% of participants. The most common forms were harassment (71.5%) and unwanted flirting (68.4%). Additionally, 19.4% had experienced female genital mutilation.

### 3.3. Causes and Severity of Violence

Table 3 demonstrates that the most commonly reported causes of violence from the participants’ perspective were lower income (69%), followed by habits and traditions (67.0%), and embedded violence (64.0%).

Regarding the severity of violence, 67% of the study sample reported that the abuser controlled the money, and 45.1% reported that their property was destroyed by the abuser. Burning of the house and pressure to have sex were reported by 11.6% and 15%, respectively. Only 4.2% were attacked with a weapon or object. More than one-third (32.2%) of the sample reported that violence had become more frequent over time.

### 3.4. Perpetrators of Violence

**Figure 1:**
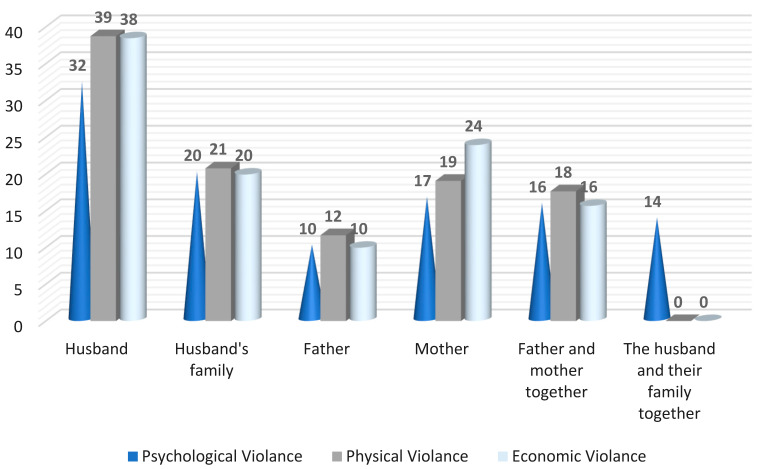
Person causing different forms of violence among the studied sample (n = 351).

Figure 1 shows that husbands were the primary perpetrators of physical (39%), psychological (32%), and economic violence (38%). The husband’s family was responsible for 21% of physical violence cases. Both psychological and economic violence were also perpetrated by the husband’s family in 20% of cases.

### 3.5. Risk Factors for Violence

**Figure 2:**
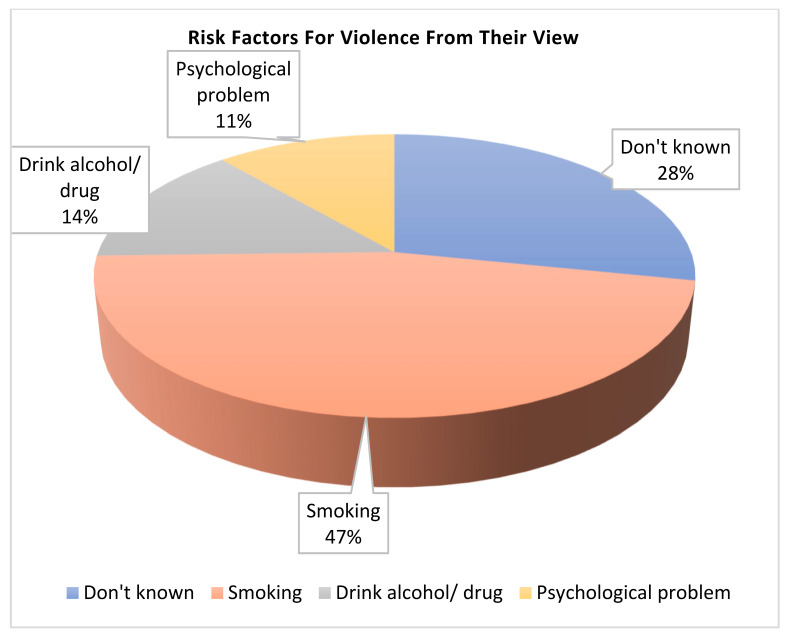
Risk factors for violence from their point of view of the studied sample (n = 379).

Figure 2 displays the risk factors for violence from the participants’ perspective. Nearly half (47%) of the abusers were cigarette smokers, 14% were alcohol and drug users, 11% had psychological problems, and approximately one-third of participants did not know any specific risk factors.

### 3.6. Consequences of Violence

**Figure 3:**
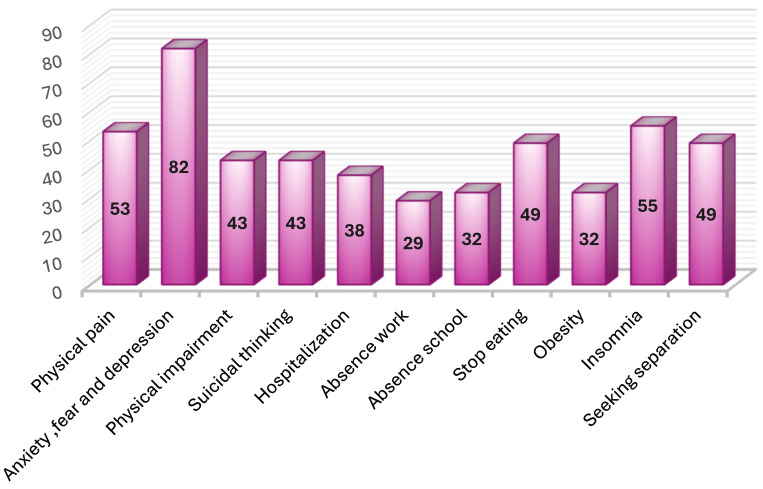
Consequences of violence among the studied sample (n = 351).

Figure 3 illustrates that the most common consequences of violence among the study sample were anxiety, fear, and depression (82%), followed by insomnia and inability to sleep (55% and 53%, respectively). Nearly half (49%) of the women reported seeking separation and stopping eating as consequences of violence.

## 4. Discussion

This study aimed to assess the prevalence, patterns, risk factors, and health sequelae of domestic violence among females of reproductive age. The findings revealed an alarmingly high prevalence of domestic violence, with significant implications for public health policy and clinical practice.

The current study found that more than one-third of females were between 29 and 39 years of age, slightly more than half were housewives, nearly half had a secondary education, and most had an average income. These findings partially align with the Bayesian analysis study by Aychiluhm et al. [15], which reported that more than half of the studied females were between 25 and 34 years old, more than half were housewives, and more than one-third were not educated. The difference may be attributed to geographical and cultural variations, as well as improved educational access in urban Egyptian settings compared to rural areas.

The present study’s finding of average income among participants is consistent with Akel et al. [16], who reported similar income levels. However, this contrasts with Agero et al. [17], who studied violence during COVID-19 and found that less than one-third had low income. Regarding obstetric history, the current study found that participants had more than three pregnancies and births, with more than one-third having experienced a previous abortion. These findings align with those of Al-Zumair et al. [18], who reported that fewer than two-thirds of the studied women had 1–5 living children.

The highly statistically significant correlation found between sociodemographic variables and different forms of violence in this study is consistent with Akel et al. [16], who found that higher total abuse scores were significantly associated with unemployment, low income, and low levels of education. This underscores the importance of socioeconomic factors as both risk factors and potential targets for intervention.

Regarding the overall prevalence and forms of violence, the present study revealed that the majority (92.6%) of studied females were exposed to violence, with more than three-quarters experiencing psychological violence, nearly two-thirds experiencing physical violence, and more than half experiencing economic and sexual violence. These findings are consistent with Derakhshanpour et al. [19], who observed that the majority of studied females were exposed to overall forms of violence. Similarly, El-Hosary et al. [20] declared that the majority of studied females were exposed to psychological violence, followed by physical and sexual violence.

The finding that husbands were the primary perpetrators of physical, psychological, and economic violence, followed by the husband’s family, is consistent with previous research in Middle Eastern contexts. Bhatta et al. [21] stated that more than half of the participants were exposed to emotional violence in the form of insults, more than one-fifth were exposed to physical violence (slapping, pushing, shaking), more than one-third to economic violence (being prevented from owning anything), and less than one-fifth to sexual violence (forced sexual intercourse).

However, these findings contrast with those of Bernards and Graham [22], who studied the cross-cultural association between marital status and physical aggression, reporting that divorced women are more likely than married women to report being victims of physical or sexual aggression by an intimate partner. This discrepancy may reflect cultural differences in reporting, divorce rates, and social support systems across different societies.

The study findings regarding causes and severity of violence align with traditional gender norms in the region. More than two-thirds of violence was attributed to lower income, followed by habits and traditions, and embedded violence. These findings partially agree with Kathryn and Bell [23], who used a behavioral analytic approach and reported that victims of violence often state they will never return to the batterer.

However, the present study findings disagree with Aidonojie et al. [24], who reported that religious and traditional beliefs of male superiority over females are a major cause of violence, followed by frustration, depression, and psychological disorders. This difference may be related to variations in cultural and religious contexts across different countries and regions.

Regarding risk factors, the present study illustrated that less than half of abusers were cigarette smokers, less than one-fifth were alcohol and drug users, and about one-tenth had psychological problems. These findings are inconsistent with Jeevasuthan and Hatta [25], who revealed that alcohol and substance abuse were the main factors for domestic violence in their study area. The difference may be attributed to cultural and religious factors, as alcohol consumption is less socially acceptable in predominantly Muslim societies like Egypt.

Furthermore, the findings disagree with Moura et al. [26], who reported that the use of alcoholic beverages by intimate partners and abuse of other drugs was considered a major risk factor for violence against women. Again, this difference may be related to cultural and religious variations across different geographical regions.

As regards the consequences of violence, the present study results illustrated that the majority of participants experienced anxiety, fear, and depression, followed by insomnia and physical pain, and then sought separation. These findings partially match those of Ceballos et al. [27], who reported that consequences of domestic violence included stress, fear, low self-esteem, emotional distress, sadness, and anxiety. The psychological sequelae of domestic violence are remarkably consistent across cultures, highlighting the universal impact of such trauma on mental health.

The high prevalence of psychological consequences found in this study underscores the importance of integrating mental health services into interventions for victims of domestic violence. Healthcare providers, particularly nurses, play a crucial role in identifying victims and providing appropriate referrals for psychological support.

From a nursing perspective, these findings emphasize the critical role of nurses in identifying and addressing domestic violence. Nurses are often the first point of contact for women seeking healthcare and are uniquely positioned to screen for violence, provide support, and facilitate referrals to appropriate services. The implementation of routine screening protocols in maternal and child health centers could significantly improve early identification and intervention.

The study’s findings have several implications for nursing practice and healthcare policy in Egypt and similar contexts. First, there is an urgent need for comprehensive training programs for nurses and other healthcare providers on recognizing signs of domestic violence and providing trauma-informed care. Second, healthcare facilities should establish clear protocols for screening, documentation, and referral of domestic violence cases. Third, multidisciplinary collaboration between healthcare providers, social services, law enforcement, and community organizations is essential for providing holistic support to victims.

Additionally, public health campaigns aimed at raising awareness about domestic violence, challenging harmful gender norms, and promoting women’s rights and empowerment are crucial. Legal enforcement of existing domestic violence legislation should be strengthened, and women should have better access to legal protection and justice services.

### Study Limitations

This study has several limitations that should be considered when interpreting the results. First, the cross-sectional design limits the ability to establish causal relationships between risk factors and violence outcomes. Second, the study was conducted only in Sharkia governorate, which may limit the generalizability of findings to other regions of Egypt or other countries. Third, self-reported data may be subject to recall bias and social desirability bias, particularly given the sensitive nature of the topic. Fourth, cultural factors and fear of stigma may have led to underreporting of certain forms of violence, particularly sexual violence. Longitudinal studies with larger, more diverse samples are needed to better understand the temporal dynamics of domestic violence and to evaluate the effectiveness of interventions.

## 5. Conclusions

The study found a high prevalence of domestic violence (88%) among reproductive-age women in Sharkia governorate, Egypt, with psychological violence being most common (78%), followed by physical (63%) and economic (43%) forms. Husbands were the main perpetrators, often linked to cigarette use, substance abuse, and psychological issues. The impact on women’s health was severe, causing anxiety, depression, fear, insomnia, and physical pain. The findings highlight the need for comprehensive, multi-sectoral interventions involving healthcare providers, especially nurses, through training, screening, and referral systems, alongside societal efforts such as awareness, empowerment, and legal enforcement. Further longitudinal research is recommended to evaluate intervention effectiveness, understand perpetrator behavior, and assess long-term outcomes for women and their children.

## Figures and Tables

**Table 1:** Correlation between sociodemographic data, clinical history, and different forms of violence in the sample studied.

General Characteristics		Total Sample		Psychological Violence (n = 269)		Physical Violence (n = 232)		Economic Violence (n = 223)		Sexual Violence (n = 211)			
		No.	%	No.	%	No.	%	No.	%	No.		%	
Female age/years													
<20		55	14.5	55	100.0	46	83.6	43	78.2	22		40.0	
20 – <29		121	32.0	81	66.9	113	93.4	105	86.8	86		71.1	
29 – <39		132	34.8	89	67.4	22	16.7	41	31.1	60		45.5	
39 – <49		71	18.7	44	62.0	51	71.8	34	47.9	43		60.6	
X^2^ (*p*-Value)				27.050 (0.0001 **)		178.104 (0.0001 **)		93.066 (0.0001 **)		23.378 (0.0001 **)			
Age at marriage/years													
Not applicable (Single)		73	19.3	73	100.0	64	87.7	61	83.6	41		56.2	
<20		90	23.7	43	47.8	54	60.0	35	38.9	46		51.1	
20 – <30		190	50.1	143	75.3	88	46.3	105	55.3	106		55.8	
30 – <40		26	6.9	10	38.5	26	100.0	22	84.6	18		69.2	
X^2^ (*p*-Value)				68.402 (0.0001 **)		55.814 (0.0001 **)		41.350 (0.0001 **)		2.704 (0.440)			
Women education													
Read and write		48	12.7	47	97.9	40	83.3	48	100.0	33		68.8	
Primary education		127	33.5	62	48.8	107	84.3	101	79.5	99		78.0	
Secondary education		134	35.3	94	70.1	52	38.8	55	41.0	61		45.5	
High education		70	18.5	66	94.3	33	47.1	19	27.1	18		25.7	
X^2^ (*p*-Value)				65.686 (0.001 **)		72.458 (0.0001 **)		102.580 (0.0001 **)		59.925 (0.0001 **)			
Place of residence													
Urban		235	62.0	178	75.7	123	52.3	133	56.6	138		58.7	
Rural		144	38.0	91	63.2	109	75.7	90	62.5	73		50.7	
X^2^ (*p*-Value)				6.827 (0.009 **)		20.511 (0.0001 **)		1.285 (0.257)		2.332 (0.127)			
Women Marital status													
Single		93	24.5	92	98.9	84	90.3	64	68.8	41		44.1	
Married		168	44.3	97	57.7	87	51.8	93	55.4	111		66.1	
Widow		27	7.1	26	96.3	11	40.7	14	51.9	13		48.1	
Divorced		91	24.0	54	59.3	50	54.9	52	57.1	46		50.5	
X^2^ (*p*-Value)				63.940 (0.0001 **)		45.752 (0.0001 **)		5.317 (0.150)		14.008 (0.003 **)			
Women occupation													
Student		55	14.5	55	100.0	46	83.6	43	78.2	22		40.0	
Housewife		194	51.2	126	64.9	177	91.2	156	80.4	140		72.2	
Working		130	34.3	88	67.7	9	6.9	24	18.5	49		37.7	
X^2^ (*p*-Value)				26.593 (0.0001 **)		246.687 (0.0001 **)		133.290 (0.0001 **)		43.887 (0.0001 **)			
Husband’s age/years													
Not applicable	74		19.5	74	100.0	65	87.8	62	83.8	41		55.4	
<30	23		6.1	14	60.9	22	95.7	19	82.6	18		78.3	
30 – <40	145		38.3	99	68.3	70	48.3	79	54.5	69		47.6	
40 – <50	79		20.8	50	63.3	35	44.3	43	54.4	55		69.6	
50 – 60	58		15.3	32	55.2	40	69.0	20	34.5	28		48.3	
X^2^ (*p*-Value)				41.211 (0.0001 **)		54.787 (0.0001 **)		40.355 (0.0001 **)		16.113 (0.003 **)			
Husband occupation													
Not applicable	74		19.5	74	100.0	65	87.8	62	83.8	41		55.4	
Work	287		75.7	190	66.2	149	51.9	149	51.9	153		53.3	
Not work	18		4.8	5	27.8	18	100.0	12	66.7	17		94.4	
X^2^ (*p*-Value)				49.742 (0.0001 **)		43.947 (0.0001 **)		25.147 (0.0001 **)		11.616 (0.003 **)			
Husband education													
Not applicable	74		19.5	74	100.0	65	87.8	62	83.8	41		55.4	
Read and write	28		7.4	27	96.4	17	60.7	8	28.6	9		32.1	
Primary education	56		14.8	32	57.1	8	14.3	9	16.1	7		12.5	
Secondary education	151		39.8	103	68.2	116	76.8	119	78.8	105		69.5	
High education	70		18.5	33	47.1	26	37.1	25	35.7	49		70.0	
X^2^ (*p*-Value)				64.130 (0.0001 **)		106.614 (0.0001 **)		112.215 (0.0001 **)		66.162 (0.0001 **)			
Income/month L. E													
1000 – <2000	13		3.4	0	0.0	13	100.0	11	84.6	12		92.3	
2000 – <4000	116		30.6	80	69.0	61	52.6	53	45.7	64		55.2	
4000 and more	159		42.0	118	74.2	67	42.1	68	42.8	67		42.1	
Does not know	91		24.0	71	78.0	91	100.0	91	100.0	68		74.7	
X^2^ (*p*-Value)				35.021 (0.0001 **)		93.901 (0.0001 **)		92.465 (0.0001 **)		32.269 (0.0001 **)			
Clinical characteristics													
No. of gravida													
Not applicable	78		20.6	74	94.9	69	88.5	66	84.6		45		57.7
<5	215		56.7	128	59.5	147	68.4	127	59.1		125		58.1
Five and more	86		22.7	67	77.9	16	18.6	30	34.9		41		47.7
X^2^ (*p*-Value)				37.288 (0.0001 **)		94.793 (0.0001 **)		41.781 (0.0001 **)			2.888 (0.236)		
No. of birth													
Not applicable	78		20.6	78	100.0	65	83.3	62	79.5		45		57.7
<5	301		79.4	191	63.5	167	55.5	161	53.5		166		55.1
X^2^ (*p*-Value)				40.161 (0.0001 **)		20.239 (0.0001 **)		17.289 (0.0001 **)			0.162 (0.687)		
No. of abortion													
Not applicable	78		20.6	78	100.0	69	88.5	62	79.5		41		52.6
<5	301		79.4	191	63.5	163	54.2	161	53.5		170		56.5
X^2^ (*p*-Value)				40.161 (0.0001 **)		30.712 (0.0001 **)		17.289 (0.0001 **)			0.385 (0.535)		
No. of living children													
Not applicable	77		20.3	77	100.0	68	88.3	63	81.8		42		54.5
<5	248		65.4	148	59.7	110	44.4	121	48.8		131		52.8
Five and more	54		14.2	44	81.5	54	100.0	39	72.2		38		70.4
X^2^ (*p*-Value)				49.749 (0.0001 **)		87.718 (0.0001 **)		31.122 (0.0001 **)			5.583 (0.061)		

** Correlation is a highly statistically significant difference at 0.01.

**Table 2:** Different forms of violence and their prevalence in the studied sample (n = 379).

Overall Prevalence of Violence	No.	%
Yes	351	92.6
No	28	7.4
Psychological violence *	269	76.6
Yelling	184	52.4
Belittling or humiliating	149	42.5
Performing things to scare him	113	32.2
Criticism and embarrassment	120	34.2
Threatened to divorce	83	23.6
Acted jealous, suspicious, and got angry if I spoke with another	88	25.1
Physical violence *	232	66.1
Slapping, throwing, beating	184	52.4
Shoving, pulling of hair	100	28.5
Kicking, dragging, or throwing things	122	34.8
Choking or burning	81	23.1
Threatening to use a knife, a weapon	65	18.5
Economic violence *	223	63.5
Against/Preventing them from working	124	35.3
Take the salary	124	35.3
Against/Preventing girls’ education	64	18.2
Participate in home expenses	120	34.2
Prevent owning anything	137	39.0
Sexual violence *	211	60.1
Flirting	240	68.4
Harassment, spiking	251	71.5
Practice sex by force	62	17.7
Rape	41	11.7
Being forced to do something sexual	78	22.2
Female genital mutilation	68	19.4

* Total is not exclusive (more than one answer).

**Table 3:** The causes of violence from the study sample’s point of view and their severity.

Causes of Violence	No.	%
Lower income	262	69.0
Habits and traditions	254	67.0
Violence is embedded	243	64.0
Illiteracy	186	49.0
Unemployment and drug use	208	55.0
The variety of social education status	171	45.0
Tolerance of violence	182	48.0
Female neglect	155	41.0
Family involvement	193	51.0
**Severity of violence**		
The abuser is destroying the property	171	45.1
The abuser controls the money	254	67.0
Burning	44	11.6
Attack with a weapon or object	16	4.2
Pressure to have sex	57	15.0
Became more frequent	122	32.2

## Data Availability

The data presented in this study are available upon request from the corresponding author due to ethical and privacy considerations.

## Public Involvement Statement

No public involvement in any aspect of this research. Members of the public did not participate in identifying the research question, designing the study, collecting data, interpreting results, or reviewing the manuscript.

## Guidelines and Standards Statement

This manuscript was prepared in accordance with the appropriate reporting guidelines for the study design. All required checklist items were addressed and integrated into the final manuscript following the journal’s standards and ethical requirements.
